# Antibiotic Profiles and Draft Genome Sequences of Kerstersia gyiorum, Providencia stuartii, Providencia vermicola, and Alcaligenes faecalis Strains Recovered from Soft Tissue Biopsy Samples in Ghana

**DOI:** 10.1128/mra.00893-22

**Published:** 2022-12-01

**Authors:** Beverly Egyir, Felicia Owusu, Christian Owusu-Nyantakyi, Grebstad Rabbi Amuasi, William Boateng, Sarkodie Kodom, Quaneeta Mohktar, Pernille Nilsson, Bright Adu, Rene S. Hendriksen

**Affiliations:** a Department of Bacteriology, Noguchi Memorial Institute for Medical Research, University of Ghana, Accra, Ghana; b Department of Immunology, Noguchi Memorial Institute for Medical Research, University of Ghana, Accra, Ghana; c Technical University of Denmark, National Food Institute, Research Group for Global Capacity Building, WHO Collaborating Centre for Antimicrobial Resistance in Foodborne Pathogens and Genomics, FAO Reference Laboratory for Antimicrobial Resistance, European Union Reference Laboratory for Antimicrobial Resistance, Kongens Lyngby, Denmark; d University of Ghana Hospital, Accra, Ghana; Montana State University

## Abstract

Whole-genome sequence data for clinically relevant Gram-negative bacteria from the African continent are scarce. In this report, we present the draft genome sequence data and antibiograms of four species, namely, Kerstersia gyiorum, Providencia vermicola, Providencia stuartii, and Alcaligenes faecalis, that were recovered from human soft tissue biopsy samples.

## ANNOUNCEMENT

Whole-genome sequencing technologies have emerged as important methodologies for diagnosis and surveillance of antimicrobial resistance (1). Here, we report on the draft genome sequences and antibiograms of four Gram-negative bacteria species, namely, Kerstersia gyiorum, Alcaligenes faecalis, Providencia stuartii, and Providencia vermicola, that were recovered from human soft tissue biopsy samples. Kerstersia gyiorum represents opportunistic bacteria (2) that are frequently resistant to chloramphenicol and ciprofloxacin (3, 4). Alcaligenes faecalis has been associated with severe clinical conditions in humans (5, 6). Providencia stuartii infections are difficult to manage (3) and are often resistant to multiple antibiotics (7–9). The NDM-1 carbapenemase gene has been detected in Providencia vermicola isolates (10, 11).

The study was approved by the Noguchi Memorial Institute for Medical Research Institutional Review Board (Ethical code number: FW00001824) and University of Ghana Ethical and Protocol Review Committee, College of Health Sciences (Protocol number: CHS-Et/M.I-P.2.13/2017-2018). Soft tissue biopsy samples that had been aseptically obtained from patients (*n* = 50) with diabetic foot ulcers were placed in 10 mL thioglycolate broth, incubated for 18 to 24 h at 37°C, and plated on 5% sheep blood agar (Oxoid, Basingstoke, UK) and MacConkey agar (Oxoid). Isolates recovered were identified to the species level using matrix-assisted laser desorption ionization–time of flight mass spectrometry (Bruker Daltonics, Germany).

The MICs for 12 antibiotics were determined with an AutoSCAN-4 system (American MicroScan, Mahwah, NJ), with the quality control strain ATCC 25922. The results were interpreted using CLSI guidelines (12).

Genomic DNA extraction and purification were performed using the QIAamp DNA minikit (Qiagen, Germany). The concentration of extracted DNA was determined using a Qubit 4.0 fluorometer. Following the manufacturer’s instructions, DNA libraries were prepared using the DNA preparation (M) tagmentation kit (Illumina Inc., San Diego, CA, USA). This process involved DNA fragmentation, tagmentation, addition of index sequences, and amplification of indexed fragments. The quality and concentration of the libraries were assessed with the 2100 Bioanalyzer system (Agilent) and quantitative PCR (qPCR) (KAPA SYBR Fast qPCR kit), respectively. Libraries were diluted to a concentration of 2 nM, pooled, and sequenced on the MiSeq platform (Illumina) using 2 × 300-bp chemistry. Indexes and reads with quality scores of <20 were trimmed using Trimmomatic v.0.39 (13), and quality control checks were performed with FastQC v.1.0. (14). Trimmed reads were assembled using Unicycler v.0.4.9 (15) and evaluated using QUAST v.5.2.0 (16). All genomes passed the basic quality metrics of Q scores of >30, minimum coverage of 20×, and minimum contig size of 200 bp. Assembled sequence data were analyzed using kmerFinder v.4.1 (https://cge.food.dtu.dk/services/KmerFinder) (17) to determine bacterial species, CARD v.3.0.9 (https://card.mcmaster.ca/analyze/rgi) (18) and ResFinder v.4.1 (https://cge.food.dtu.dk/services/ResFinder) (19) to identify resistance genes, PlasmidFinder v.2.1 (https://cge.food.dtu.dk/services/PlasmidFinder) (20) to detect plasmids, VirulenceFinder v.2.0 (https://cge.food.dtu.dk/services/VirulenceFinder) (21) to detect virulence genes, and multilocus sequence typing (MLST) v.2.0 (https://cge.food.dtu.dk/services/MLST) (22) to determine sequence types. The draft genomes were annotated using Prokka v.1.14.6 (23). Table 1 summarizes the results.

**TABLE 1:** Antimicrobial resistance profiles and genomic characteristics of isolates

Characteristic	Data for^*a*^:			
	Providencia stuartii	Kerstersia gyiorum	Providencia vermicola	Alcaligenes faecalis
MIC (mg/L)				
Amikacin	≤16 (S)	≤16 (S)	>32 (R)	≤16 (S)
Aztreonam	8 (S)	8 (S)	>16/8 (R)	>8 (R)
Cefepime	8 (S)	4 (S)	>8 (R)	>8 (R)
Ceftazidime	4 (S)	≤1 (S)	NT	>16 (R)
Ciprofloxacin	>2 (R)	>2 (R)	>2 (R)	>2 (R)
Gentamicin	8 (I)	≤4 (S)	>8 (R)	>8 (R)
Imipenem	4 (S)	≤1 (S)	>8 (R)	≤1 (S)
Levofloxacin	>4 (I)	≤2 (S)	>4 (R)	>4 (R)
Meropenem	≤1 (S)	≤1 (S)	>8 (R)	≤1 (S)
Piperacillin-tazobactam	>64 (R)	≤16 (S)	>64 (R)	>64 (R)
Tobramycin	>8 (R)	≤4 (S)	>8 (R)	>8 (R)
Trimethoprim-sulfamethoxazole	>2/38 (R)	≤2/38 (S)	NT	>2/38 (R)
Resistance genes				
Aminoglycosides	*aac(2′)-Ia*, *aadA1*, *aph(3′)-Ia*	ND	*aac(6')-Ib-cr*, *aph(3′)-VI*, *aadA1*, *rmtC*, *ant(3″)-IIa*	*aac(6')-Ib-cr*, *aac(6')-Ib3*, *aadA1*, *ant(2″)-Ia*, *aph(3″)-Ib*, *aph(6)-Id*
Sulfonamides	*sul3*	ND	*sul1*	*sul1*
β-Lactams	ND	ND		
Cephalosporins			*bla*_DHA-1_	
Carbapenems			*bla*_OXA-10_, *bla*_NDM-1_	*bla*_CARB-4_
Tetracyclines	*tet*(B)	ND	ND	*tet*(A), *tet*(G)
Diaminopyrimidines	*dfrA1*	ND	*dfrA14*	*dfrA15*, *dfrA17*
Fluoroquinolones	*qnrD1*	ND	*qnrA1*, *aac(6′)-Ib-cr*	*aac(6')-Ib-cr*
Phenicols	*catA3*	*catB3*	ND	*catB3*, *floR*
Rifamycins	ND	ND	*arr-2*	ND
Disinfecting agents and antiseptics	*qacL*, *rsmA*	ND	*qacE*	*qacE*
Macrolides	*crp*	ND	ND	ND
Nucleosides	*sat-2*	ND	ND	ND
Genome size (bp)	4,329,306	3,983,113	4,455,003	4,464,976
Sequence type	Unknown	Unknown	Unknown	Unknown
*N*_50_ (bp)	187,893	485,120	642,172	282,357
GC content (%)	41.2	62.5	40.8	56.8
No. of reads	808,942	621,880	782,484	989,950
No. of contigs	106	17	63	66
Size of largest contig (bp)	650,510	1,055,846	1,087,565	915,131
No. of coding sequences	3,990	3,466	3,988	4,191
No. of rRNAs	4	3	5	3
No. of tRNAs	71	52	70	52
No. of transfer-messenger RNAs	1	1	1	1
Coverage (×)	38	26	35	41
Genome accession no.	JANKKP000000000	JANKLF000000000	JANKLM000000000	JANKKY000000000
BioSample accession no.	SAMN29222022	SAMN29222006	SAMN29221999	SAMN29222013
SRA accession no.	SRR21354960	SRR21354962	SRR21354959	SRR21354961

a NT, not tested; R, resistant; I, intermediate; S, susceptible; ND, not detected. No virulence genes were determined for all organisms.

### Data availability.

Assembled data were deposited in the National Center for Biotechnology Information (NCBI) database under the BioProject accession number PRJNA851374. This whole-genome shotgun project was deposited in DDBJ/ENA/GenBank under the accession numbers JANKKP000000000, JANKLF000000000, JANKLM000000000, and JANKKY000000000, as listed in Table 1.
